# Development of a vaccine based on mRNA assembly of PEDV virus-like particle

**DOI:** 10.1128/jvi.02060-25

**Published:** 2026-04-21

**Authors:** Mengdi Yang, Yongxiang Zhao, Weilu Guo, Lizhong Wang, Xu Song, Xinmei Geng, Shiyu Liu, Hongqi Shang, Mi Hu, Shanshan Yang, Yunchuan Li, Min Sun, Lixiang Zhao, Tianyi Zhong, Bin Li, Baochao Fan

**Affiliations:** 1 Institute of Veterinary Medicine, Jiangsu Academy of Agricultural Sciences, Key Laboratory of Veterinary Biological Engineering and Technology, Ministry of Agriculture; Jiangsu Key Laboratory for Food Quality and Safety-State Key Laboratory Cultivation Base of Ministry of Science and Technology 668638 , Nanjing, China; 2 College of Veterinary Medicine, Nanjing Agricultural University 261674, Nanjing, China; 3 Taizhou Polytechnic College 164417 , Taizhou, China; 4 Suzhou Huiliao Biomedical Technology Co., Ltd., Suzhou, China; 5 Suzhou Medical College, Soochow University 12582, Suzhou, China; 6 Jiangsu Co-innovation Center for Prevention and Control of Important Animal Infectious Diseases and Zoonoses, Yangzhou University 38043, Yangzhou, China; 7 GuoTai (Taizhou) Center of Technology Innovation for Veterinary Biologicals, Taizhou, China; 8 School of Food and Biological Engineering, Jiangsu University 506405, Zhenjiang, China; University of North Carolina at Chapel Hill, Chapel Hill, North Carolina, USA

**Keywords:** PEDV, mRNA vaccine, virus-like particle, immunogenicity

## Abstract

Porcine epidemic diarrhea virus (PEDV) represents a highly contagious enteropathogenic coronavirus affecting swine. This virus elicits acute diarrhea in neonatal nursing piglets, with mortality approaching 100%, and, thus, imposes enormous economic burdens on the worldwide pork industry. Therefore, developing safe and effective vaccines remains a top priority for controlling PEDV. Here, we constructed a lipid-nanoparticle (LNP)-encapsulated messenger RNA (mRNA) vaccine. This vaccine encodes the four major structural proteins of PEDV: spike (S), membrane (M), envelope (E), and nucleocapsid (N), which self-assemble into virus-like particle (VLP). Compared with a PEDV S-only mRNA vaccine and a commercial PEDV/transmissible gastroenteritis virus (TGEV) bivalent inactivated vaccine in a mouse model, this VLP mRNA vaccine induced significantly higher levels of IgG, mucosal IgA, and neutralizing antibodies. It also enhanced the lymphocyte proliferation index and expanded the populations of T and B cells. Subsequent evaluations in sows showed that the VLP mRNA vaccine elicited robust PEDV-specific immune responses *in vivo*. Furthermore, piglets that suckled colostrum from these vaccinated sows gained protection against challenge with a virulent PEDV strain. These protected piglets exhibited lower diarrhea scores, reduced viral shedding, less severe intestinal lesions, and decreased mortality. Collectively, these findings demonstrate that the developed PEDV VLP mRNA platform constitutes a highly promising strategy for preventing PEDV infections.

**IMPORTANCE:**

PEDV-induced watery diarrhea in neonatal piglets remains a major threat to the global swine industry, while current commercial vaccines are predominantly inactivated formulations or S-only subunit vaccines, whose efficacy and broad-spectrum protection continue to be limited. We, therefore, incorporated the four PEDV structural proteins (S-M-E-N) into a LNP mRNA vaccine that self-assembles into VLPs *in vivo*. In mice, the mRNA-VLP platform elicited significantly higher systemic IgG, mucosal IgA, and neutralizing antibody titers than an S-only mRNA control or a commercial PEDV/TGEV inactivated vaccine, while expanding both T- and B-cell populations. Maternal immunization of pregnant sows converted these responses into potent systemic immunity that protected suckling piglets against virulent challenge, as evidenced by markedly reduced diarrhea scores, intestinal lesions, viral shedding, and mortality. Collectively, the developed PEDV mRNA-VLP platform offers a highly promising strategy for preventing PEDV infection.

## INTRODUCTION

PEDV is classified within the Alphacoronavirus genus of the family Coronaviridae. It is an enveloped virus with a positive-sense single-stranded RNA genome (1). The PEDV genome is about 28 kb in length and contains seven open reading frames (ORFs). These ORFs encode a set of viral proteins, including the replicase polyproteins 1a and 1ab, spike (S) protein, ORF3, envelope (E) protein, membrane (M) protein, and nucleocapsid (N) protein (2–4). The coronavirus spike protein is a multifunctional protein responsible for mediating viral entry into host cells. It initially binds to cellular receptors through the S1 subunit and subsequently drives the fusion between viral and host membranes via the S2 subunit (5). The coronavirus E protein is a small integral membrane protein that serves a critical function in the process of viral assembly. It has been shown that the functions of E protein during infection are not limited to assembly but also include viral release and host stress response (6). The M protein plays a central role in virion morphogenesis. The M protein mediates the organization of viral membrane components, and its self-interaction as well as association with the N protein promote viral assembly and budding (7). Coronavirus N proteins bind to genomic RNA to form ribonucleoprotein complexes, interact with viral membrane proteins during assembly, and play a critical role in enhancing viral transcription and assembly efficiency (8).

PEDV was first identified in Europe during the early 1970s. After 2010, highly pathogenic variant strains of PEDV have spread widely among pig herds, resulting in considerable economic losses on a global scale (9, 10). Vaccine immunization is still an effective method to control the epidemic of PEDV. Currently, a variety of PEDV single vaccines or multiple vaccines containing PEDV are available, but conventional inactivated or attenuated vaccines are the main ones (11, 12). The immune efficacy of inactivated vaccines is often weak, while attenuated vaccines require long development periods and carry the risk of re-intensification of virulence and recombination with circulating strains (13). It is crucial to develop new and efficient PEDV vaccines.

mRNA vaccines are characterized by potent immunogenicity and high effectiveness. The mRNA technology facilitates the rapid design and development of vaccines targeting multiple antigens and can serve as a self-adjuvant to trigger innate immune responses (14, 15). Furthermore, we have previously successfully developed a PEDV mRNA vaccine based on the S protein, which demonstrated favorable immunoprotective efficacy (16). Compared with traditional subunit vaccines, VLPs exhibit prominent advantages owing to their bigger size and repetitive antigen presentation, enabling robust stimulation of both innate and adaptive immune responses (17, 18). Constructing VLPs via the mRNA platform may further enhance the vaccine’s immunogenicity and protective efficacy. Moreover, the VLP-type mRNA vaccines against HIV-1 and SARS-CoV-2 have also been reported (19, 20).

Accordingly, we designed an LNP-encapsulated VLP mRNA vaccine encoding PEDV S, M, E, and N proteins. Following immunization in mice, this VLP mRNA vaccine showed superior immunogenicity compared with the S mRNA vaccine and the PEDV/TGEV bivalent inactivated vaccine. Furthermore, subsequent immunization of pregnant sows and challenge experiments in neonatal piglets confirmed that this VLP mRNA vaccine provides a high level of protective efficacy.

## MATERIALS AND METHODS

### Cells and viral strains

HEK-293T cells were maintained in Dulbecco’s modified Eagle’s medium (DMEM) supplemented with 15% (vol/vol) heat-inactivated fetal bovine serum (FBS) at 37°C in a 5% CO₂ atmosphere. The Vero cell line (American Type Culture Collection CCL-81) was used for PEDV propagation and cultured in DMEM supplemented with 10% (vol/vol) heat-inactivated FBS at 37°C in 5% CO₂. PEDV strains CH-HK-2021 (GenBank no. PP785988, G2c subtype), AH2012/12 (GenBank no. KU646831, G2b subtype), JS2008 (GenBank no. KC210146, G1 subtype), and JS-NJ2023 (GenBank no. PP580804, S-INDEL subtype) (Fig. S1) were isolated, maintained in our laboratory, and cultured in Vero cell culture medium supplemented with 5 µg/mL trypsin and 37.5 µg/mL pancreatin.

### mRNA production and LNP encapsulation

**Fig 1:**
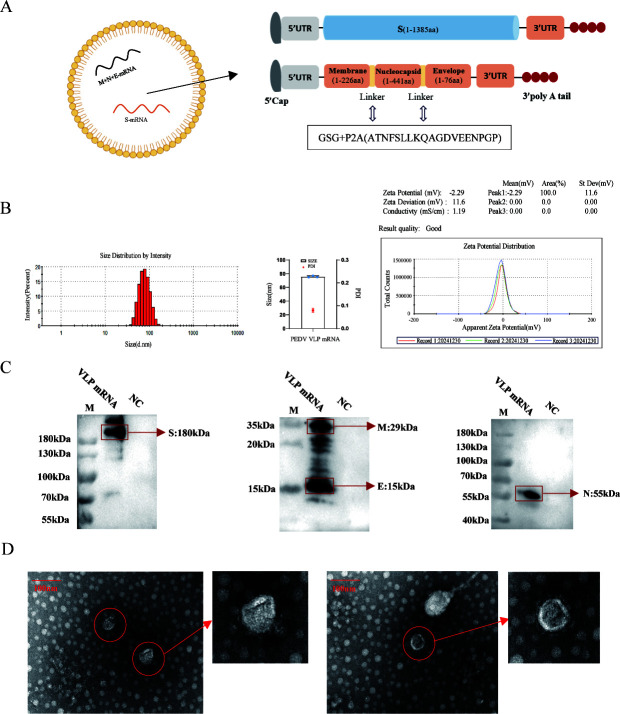
mRNA vaccine characterization and PEDV VLP identification. (**A**) Schematic diagram of mRNA vaccine design. (**B**) Size distribution, polydispersity index (PDI), and Zeta potential profile of PEDV VLP mRNA vaccine. The antigen expression of VLP mRNA in HEK293T cells was analyzed by WB (**C**) and observed by electron microscopy (**D**).

Based on the structural protein sequence of the PEDV variant CH-HK-2021, codon-optimized open reading frames were chemically synthesized. To enable simultaneous expression of all four structural proteins, one recombinant plasmid was constructed to carry the full-length S gene, whereas a second plasmid carried a single polycistronic cassette encoding M, N, and E proteins linked in frame by flexible glycine-serine-glycine (GSG) spacers together with a self-cleaving P2A peptide (21, 22). Both recombinant plasmids included coding sequences for the type I cap structure (N7MGPPPAM) and 5′ and 3′ untranslated regions (UTRs). S- and M+N + E-encoding mRNA were synthesized via *in vitro* transcription using T7 RNA polymerase and the linearized plasmid DNA templates (containing the optimal codon for the structural proteins). An equimolar mixture of S-mRNA and M+N + E-mRNA (1:1) was prepared and encapsulated into lipid nanoparticles using a microfluidic device (MaiAna I Nano L) by mixing the lipid-ethanol phase with the aqueous mRNA phase at a volumetric ratio of 1:3, yielding the PEDV VLP mRNA vaccine (Fig. 1A). The lipid formulation of the mRNA vaccine comprised SM102, 1,2-distearoyl-sn-glycero-3-phosphocholine (DSPC), cholesterol (CHOL), and DMG-PEG2000, with the detailed molar ratio of the lipid components as follows: SM102:DSPC:CHOL:DMG-PEG2000 = 50:10:38.5:1.5. The particle size, polydispersity index (PDI), and zeta potential of the prepared formulation were measured as previously described (16).

### Preparation of PEDV-positive swine hyperimmune sera

The CH-HK-2021 strain was inactivated with β-propiolactone and mixed with Gel-02 adjuvant at a 4:1 (vol/vol) ratio to prepare the inactivated vaccine. One-month-old PEDV antibody-negative piglets were selected, and each piglet received 2 mL of the corresponding inactivated vaccine intramuscularly. Booster immunizations were given at 14 and 28 days post-primary vaccination (dpv). Blood samples were collected at 42 dpv, and serum was isolated for western blotting (WB) analysis.

### VLP purification

PEDV VLP mRNA were directly added to HEK-293T cells cultured in T75 flasks without any transfection reagent. Cell supernatants were collected after 48 h of incubation; PEDV VLPs were collected after ultrasonic lysis, followed by low-speed centrifugation to remove cell debris and filtration through a 0.45 μm filter (Port Washington, New York, USA). Subsequently, the supernatants were centrifuged at 30,000 rpm and 4°C for 2 h to collect the pellets. After purification via a 30%–40%–50% discontinuous sucrose gradient, the PEDV VLP bands at the 30%–40% sucrose interface were collected, resuspended in PBS, and centrifuged again at 30,000 rpm and 4°C for 2 h to remove sucrose. Finally, the resulting pellets were resuspended in PBS and stored at −80°C.

### Electron-microscopic characterization of VLPs

Following the established experimental protocol (23), further processing was performed on the purified VLPs. For negative-stain transmission electron microscopy (TEM), 10 µL of purified PEDV VLPs was adsorbed onto carbon-coated 400-mesh copper grids for 5 min at ambient temperature. Excess sample was blotted away with Whatman No. 1 filter paper, and the grids were immediately stained with 1% (wt/vol) sodium phosphotungstate (pH 7.0) for 5 min. After a second blotting step to remove residual stain, the grids were air-dried and visualized on a JEM-1400 transmission electron microscope operating at 120 kV.

### Western blotting

PEDV VLP mRNA were directly added to HEK-293T cells cultured in T25 flasks without any transfection reagent, and the cell supernatants were collected after 24 h of incubation. Proteins were resolved by SDS-PAGE and electrotransferred onto 0.22 µm PVDF membranes (Millipore, Bedford, MA, USA). Membranes were blocked with 5% (wt/vol) skim milk in PBST (PBS supplemented with 0.05% Tween 20, pH 7.4) for 1 h at 37°C and then incubated for 2 h at 37°C with the following primary antibodies: porcine hyperimmune serum against PEDV (1:250, used for detection of M and E proteins); mouse anti-PEDV S monoclonal antibody (1:5,000); and mouse anti-PEDV N monoclonal antibody (1:5,000) all prepared in our laboratory. After three 15-min washes with PBST, membranes were probed with HRP-conjugated secondary antibody (1:10000) for 1 h at 37°C. Following three additional PBST washes, immunoreactive bands were visualized using an enhanced chemiluminescence system (Tanon 5200, Shanghai, China) and digitally captured.

### Design of the mouse vaccination experiments

**Fig 2:**
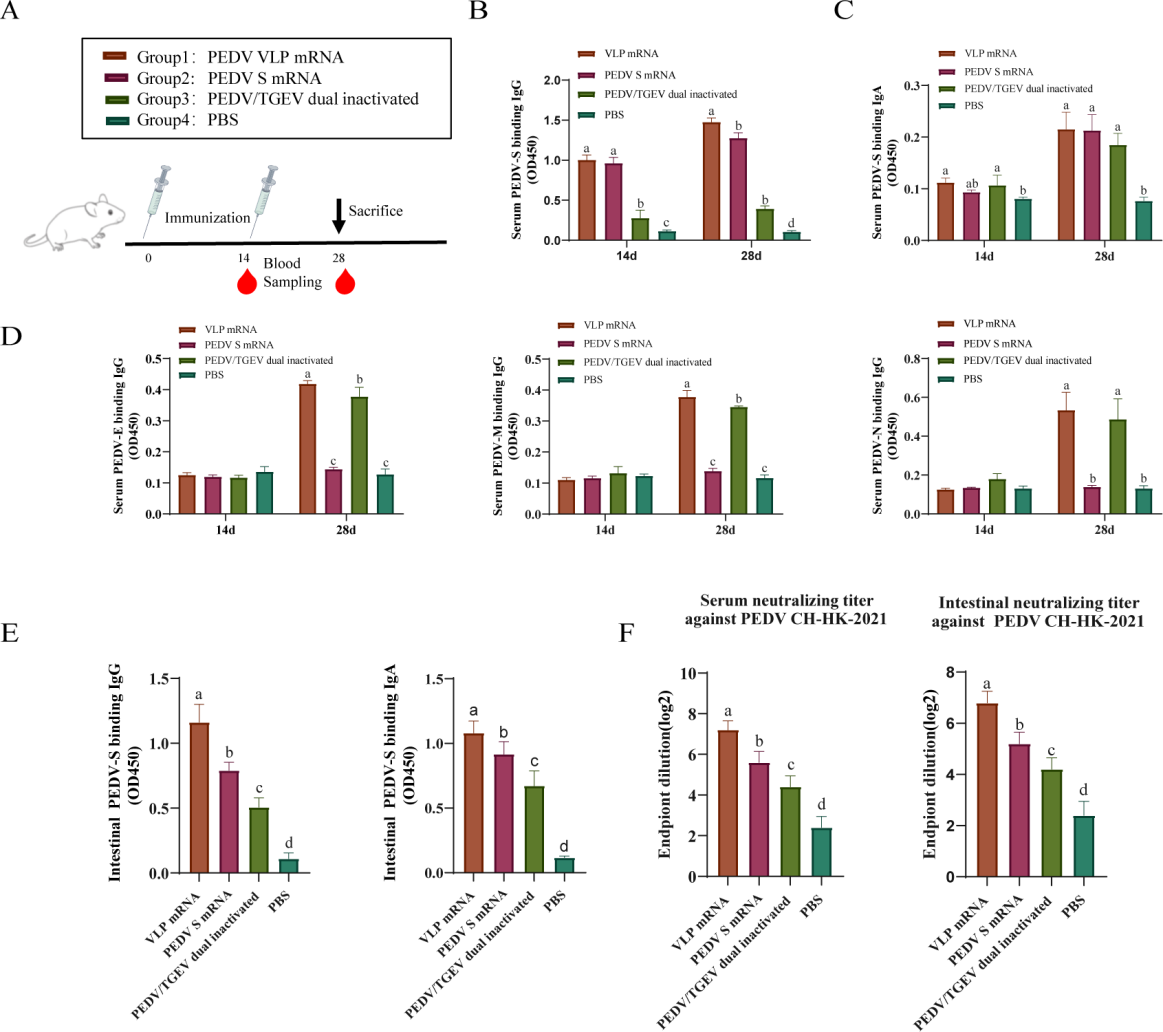
Determination of antibody levels against different structural proteins of PEDV in mouse serum and intestinal samples. (**A**) Mouse immunization program. The levels of PEDV S-specific IgG (**B**) and IgA (**C**) in serum were determined by ELISA. (**D**) PEDV E, M, and N-specific IgG levels in serum were determined by ELISA. (**E**) PEDV S-specific IgA and IgG levels of the intestinal samples were measured by ELISA. (**F**) Neutralizing antibody titers in serum samples. Data were expressed as mean ± SD of five mice in each group. One-way ANOVA and Tukey’s multiple comparison test were used. Differences between groups were significant when *P* < 0.05, a>b > c>d > e.

The immunization schedule is shown in Fig. 2A. Twenty 6-week-old female BALB/c mice were randomly divided into four groups (*n* = 5). According to previous studies, 100 µL of commercial inactivated vaccine and 10 µg of mRNA-LNP vaccine were sufficient to elicit effective immune responses in mice (16, 23, 24). So, in this study, the experimental groups received an intramuscular injection of 10 µg of mRNA-LNP vaccine or 100 µL of commercial inactivated vaccine on days 0 and 14, while the control group received an equal volume of sterile PBS (100 µL). All mice were euthanized 14 days after the last immunization (i.e., on day 28). Orbital blood was collected on days 14 and 28, and serum anti-S, E, M, and N-specific IgG levels were determined by ELISA (enzyme-linked immunosorbent assay), neutralizing antibody titers were measured by neutralization assay (25). Spleens were aseptically removed to prepare single-cell suspensions, and the percentages of CD3^+^CD4^+^, CD3^+^CD8^+^ T cells, and B220^+^CD19^+^ B cells were analyzed by flow cytometry. Splenocytes were restimulated with PEDV virions for 72 h, after which lymphocyte proliferation was assessed, the culture supernatants were collected and IFN-γ and IL-4 concentrations were quantified using a mouse IFN-γ and IL-4 ELISA kit (Meimian, China) according to the manufacturer’s instructions.

### Flow cytometry

Lymphocyte proliferation assays were performed by using splenocytes as described before (24). On day 28 dpv, splenic lymphocytes were aseptically isolated, transferred to 1.5 mL centrifuge tubes (1 × 10⁶ cells/tube), washed once with PBS, and centrifuged at 1,500 rpm for 5 min. The cell pellets were resuspended in 300 µL PBS containing the following fluorochrome-conjugated antibodies: PE anti-mouse CD4 (BioLegend, USA), APC anti-mouse CD8a (BioLegend, USA), FITC anti-mouse CD3e (BD Biosciences, USA), PerCP-Cy5.5 anti-mouse B220 (BD Biosciences, USA), and FITC anti-mouse CD19 (BioLegend, USA). After incubation at 4°C for 30 min in the dark, cells were centrifuged at 1,500 rpm for 5 min; the supernatants were discarded, and the pellets were rinsed twice with PBS. Cells were finally resuspended in 500 µL PBS and analyzed on an Accuri C6 Plus flow cytometer (BD Biosciences); 1 × 10⁵ events were acquired to enumerate CD4^+^ and CD8^+^ T cells. Data were processed with FlowJo v10.7.1 software.

### Design of the pig vaccination and viral challenge experiments

In the passive immunization and challenge trial, six PEDV-uninfected pregnant sows were selected and intramuscularly injected with either PEDV VLP mRNA vaccine (100 µg per sow) or an equivalent volume of PBS at 1 month before farrowing; the vaccinated sows received a booster dose of the same amount 14 days later. On the day of farrowing, serum and colostrum samples were collected from each sow for antibody testing. After 5 days of lactation, five neonatal piglets from each sow were randomly chosen and orally challenged with the PEDV CH-HK-2021 strain at 2 × 10^4^ TCID₅₀ per piglet. Rectal swabs and Fecal consistency scores were collected daily post-challenge. At 13 days post-challenge (dpc), the piglets were euthanized and necropsied; small-intestinal tissues and contents were collected for histopathological examination and PEDV RNA load quantification, respectively.

### ELISA

ELISA was performed to quantify IgG and IgA antibodies specific for the PEDV-S, E, M, and N. As described before (26), recombinant eukaryotic PEDV-S, E, and M proteins and prokaryotically expressed N proteins (100 ng/well) were coated in wells of a 96-well plate, suspended in bicarbonate buffer (100 mM, pH 9.6), and incubated at 4°C overnight. The coating solution was discarded, washed three times with PBST, and incubated with 3% BSA for 2 h at 37°C. The addition of serum or colostrum collected from mice or sows to be tested (1:50), after incubation for 30 min at 37°C, the wells were washed three times with PBST. Then, 100 µL of goat anti-pig/mouse IgG or goat anti-pig/mouse IgA [both HRP conjugates] was added to each well and incubated at 37°C for 30 min. Then, 100 µL of TMB substrate solution was added to each well and incubated at 37°C for 15 min. Finally, the reaction was stopped by adding 50 µL of H_2_SO_4_ (2 M) to each well, and the absorbance at 450 nm (OD_450_) was measured.

### Evaluation of neutralizing antibody titers

As described before (16), porcine and mouse serum were heat-inactivated at 56°C for 30 min, whereas intestinal tissues were directly homogenized and centrifuged to collect the supernatants. They were then serially twofold diluted, mixed with an equal volume of PEDV (200 TCID_50_/100 µL), and incubated at 37°C for 1 h. Monolayers of Vero cells in a 96-well tissue culture plate were then inoculated with the mixture. After incubation at 37°C for 2.5 h, the mixture was discarded, and the wells were washed three times with DMEM. DMEM trypsin (10 µg/mL), which is necessary for the virus to enter the cells, was then added to each well, and the cells were incubated at 37°C for 3–5 days.

### Evaluation of clinical signs and gross/histological lesions

Following the viral challenge, the piglets were monitored daily for clinical signs of diarrhea. Diarrhea was assessed by scoring fecal consistency. Fecal consistency was scored on a 0–3 scale: 0, well-formed, normal feces; 1, soft and pasty but still shaped; 2, semi-liquid diarrhea with scant solid residue; 3, watery diarrhea without any formed matter. Piglets with a score ≥2 were defined as diarrheic (27). At necropsy, tissues from the jejunum and ileum were collected and fixed in formalin for histological examination. The dehydrated tissues were treated with xylene, embedded in paraffin wax, sliced, and mounted on slides. The slides were subjected to hematoxylin and eosin (H&E) staining and an immunohistochemistry (IHC) assay. PEDV antigen was detected in fixed tissue sections by IHC using our laboratory-produced PEDV-specific monoclonal antibody (1:200 dilution) according to the protocol described before (28). Additionally, intestinal tissue samples were collected from piglets for IgA quantification.

### Quantification of PEDV RNA by quantitative real-time PCR

Viral RNA copies in anal swabs and intestinal tissues of piglets were quantified by TaqMan real-time RT-qPCR (29). Total RNA was extracted from 10% (wt/vol) suspensions of swabs or homogenized intestinal mucosa, reverse-transcribed using a commercial kit (Vazyme, China), and amplified with Taq Pro HS Probe Master Mix (Vazyme) according to the manufacturer’s protocol. Primer–probe sequences targeting the PEDV N gene were: forward, 5′-GTCTGAAAAGCCAATCATTC-3′; reverse, 5′-TTGCCTCTGTTGTTACTC-3′; probe, 5′-CTGTTGTTGCCATTGCCACGA-3′.

### Statistical analyses

The obtained data were plotted using GraphPad Prism version 8.0 for Windows (version 11; GraphPad Software Inc), and statistical analysis was performed by one-way ANOVA or two-way ANOVA, followed by Tukey’s multiple comparisons test for comparisons among groups. The data are presented as Mean ± SD, and the significance of differences between groups was indicated by letters and asterisks. The number of letters (a, b, c, d, e) was only used to distinguish the hierarchy of differences among multiple groups and had no corresponding relationship with specific *P*-value ranges: groups labeled with the same letter indicated no significant difference between them, while groups labeled with different letters indicated a significant difference between them (at *P*<0.05, the order of difference levels was a>b > c>d > e). The asterisk annotation criteria were as follows: *P < 0.05; **P < 0.01; ***P < 0.001; ****P < 0.0001; ns, no significant difference.

## RESULTS

### mRNA vaccine characterization and PEDV VLP identification

The preparation strategy of PEDV VLP mRNA is illustrated in Fig. 1A. All formulated mRNA-LNP batches exhibited average particle diameters of approximately 75 nm and polydispersity indices below 0.1; the zeta potential was approximately −2.29 mV, and the encapsulation efficiency exceeded 90% (Fig. 1B), all of which collectively demonstrate high formulation quality and stability of the VLP mRNA vaccine.

To determine the expressions of PEDV S, E, M, and N proteins, the VLP mRNA were transfected into HEK293T cells, and WB assay showed the expressions of S, N, M, and E proteins at sizes of approximately 180, 55, 29, and 15 kDa, respectively (Fig. 1C), which revealed that the four structural proteins of PEDV were expressed successfully. To explore whether the co-expressed S, N, E, and M proteins could assemble into VLPs, the purified VLP samples were observed by TEM. TEM images showed that the VLPs exhibit the spike structures, with a diameter of approximately 100 nm, and are similar to coronavirus particles in terms of characteristics (Fig. 1D).

### Immunogenicity of PEDV VLP mRNA vaccines in mice

Mice were immunized with the PEDV VLP mRNA, S mRNA, and the dual inactivated vaccines according to the schedule shown in Fig. 2A. Throughout the study, no adverse reactions were observed. ELISA results (Fig. 2B and C) revealed that after two immunizations, serum IgG and IgA antibodies specific for PEDV-S increased progressively; the IgG and IgA titers in the VLP mRNA and S-mRNA groups were significantly higher than those in the PEDV/TGEV dual-inactivated vaccine and PBS control groups. In addition, serum IgG titers directed against E, M, and N proteins in the VLP mRNA and inactivated vaccine immunized groups were also significantly increased after the second immunization (Fig. 2D). Analyses of intestinal contents showed that IgG and IgA levels in the VLP mRNA group were significantly higher than those in all other experimental groups (Fig. 2E). The neutralizing antibody titers to the PEDV CH-HK-2021 strain of serum and intestinal contents in the VLP mRNA group reached approximately 1:2^7^, which was significantly higher than those in the S mRNA-immunized and inactivated-vaccine-immunized groups (Fig. 2F).

**Fig 3:**
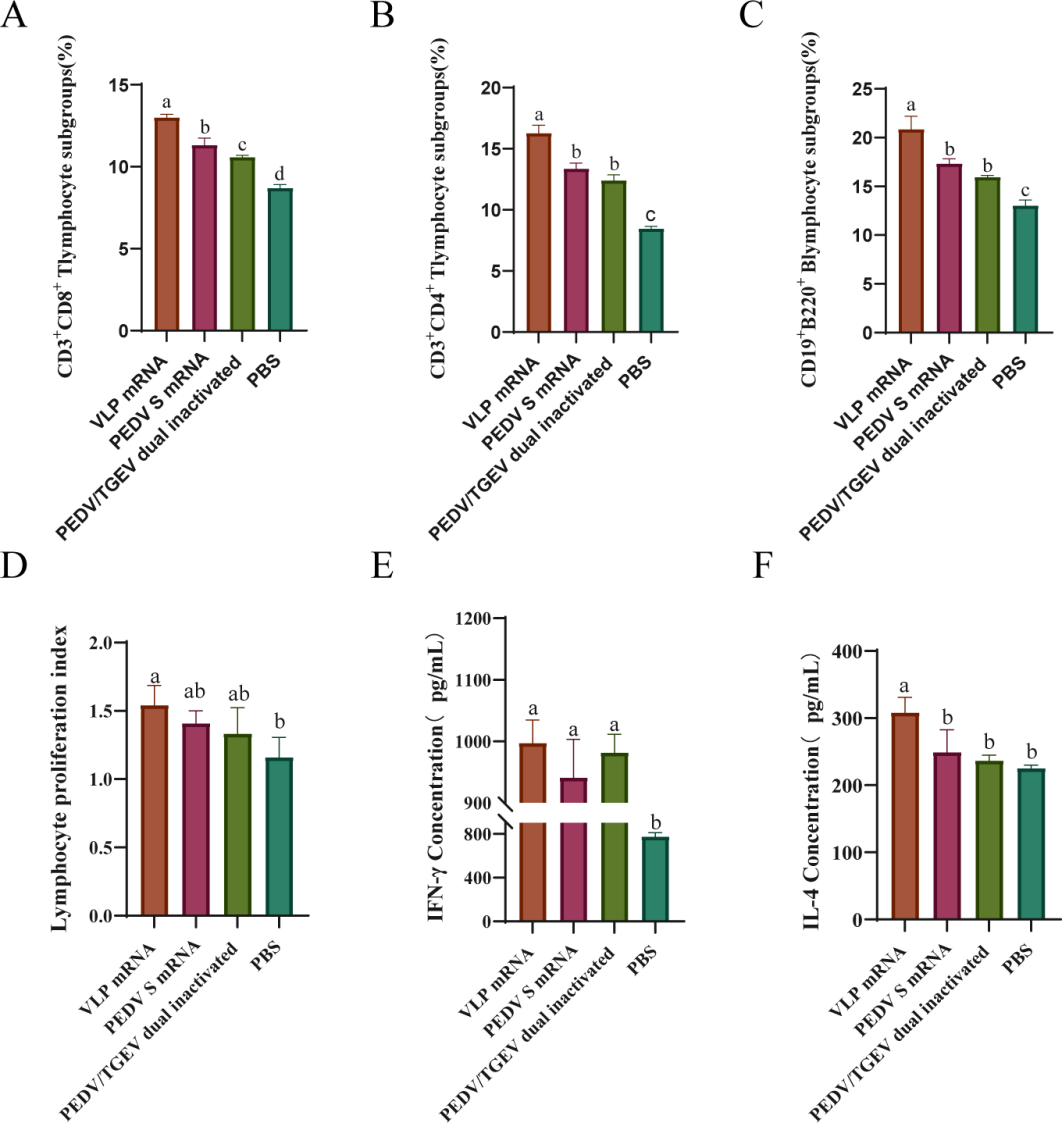
Determination of B- and T-lymphocyte subpopulations in mouse splenic lymphocytes. Flow cytometry was used to analyze the proportions of CD3^+^ CD8^+^ T (**A**), CD3^+^ CD4^+^ T (**B**), and CD19^+^ B220^+^ B (**C**) cells in splenocytes from different groups of immunized mice. Lymphocyte proliferation (**D**), IFN-γ (**E**), and IL-4 (**F**) secretions were detected in different vaccine immunized groups. Data are means ± SD for each group. One-way ANOVA and Tukey’s multiple comparison test were used. Means with different lowercase letters are statistically significant (*P* < 0.05), a>b > c>d.

To detect the immune response induced by the VLP mRNA vaccine, mouse splenic lymphocytes were isolated at 28 dpv, and B- and T-lymphocyte subpopulations were characterized and counted by flow cytometry. The results showed that the percentages of CD3^+^CD8^+^ (Fig. 3A), CD3^+^CD4^+^ (Fig. 3B) T cells, and CD19^+^B220^+^ B lymphocytes (Fig. 3C) in the VLP mRNA group were higher than those in the S mRNA, inactivated vaccine, and the PBS control groups. The lymphocytes were re-stimulated *in vitro* with the PEDV viral solution (1:10) to analyze the antigen-specific proliferative response of the lymphocytes. VLP mRNA immunized group showed the highest stimulation index (Fig. 3D). To further characterize the cellular immune response induced by the VLP mRNA vaccine, we measured IFN-γ and IL-4 secreted by virus restimulated lymphocytes by ELISA (Fig. 3E and F). The results showed that the levels of IFN-γ in three vaccines immunized mice were all significantly increased. However, the level of IL-4 was only increased in the VLP mRNA immunized group. All data revealed that the VLP mRNA vaccine induces higher levels of humoral and cellular immunity.

### Characterization of the antibody response elicited by the PEDV VLP mRNA vaccine in sows

**Fig 4:**
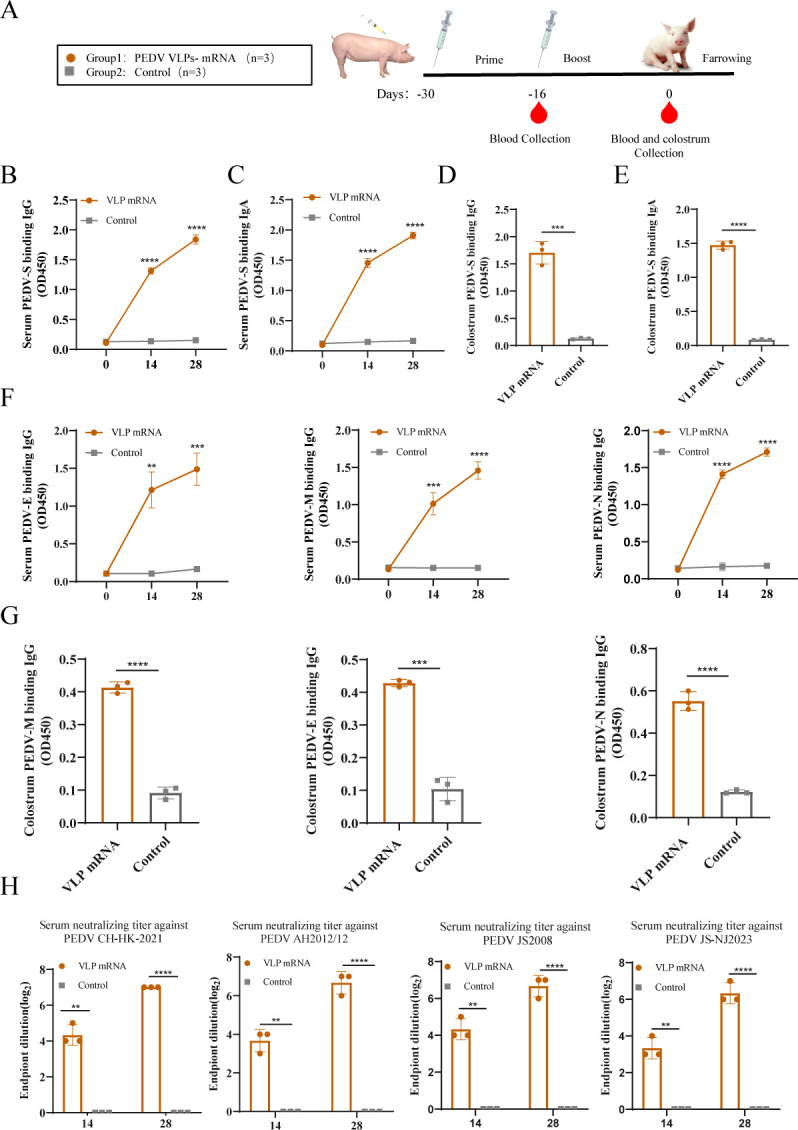
Characterization of the antibody response elicited by PEDV VLP mRNA vaccines in sows. (**A**) Immunization program of pregnant sows. Sows were vaccinated with VLP mRNA vaccine (group 1) or PBS as control (group 2) at two time points, 30 days and 16 days before farrowing (set as day −30 and day −16). Serum samples were collected on days −16 and 0, and colostrum was collected on the day of delivery. Levels of IgG and IgA antibodies bound to PEDV S protein were determined in serum (**B and C**) and colostrum (**D and E**). Serum and colostrum PEDV E, M, and N-specific IgG levels were determined by ELISA (**F and G**). Detections of the serum neutralizing antibodies against PEDV CH-HK-2021, AH2012/12, JS2008, and JS-NJ2023 (**H**). Data were expressed as mean ± SD of each group, **P < 0.01, ***P < 0.001, ****P < 0.0001.

In the passive immunization study section, we further evaluated the immunogenicity of the PEDV VLP mRNA vaccine in pregnant sows. For this purpose, we vaccinated six pregnant sows with either the VLP mRNA vaccine or PBS and received a booster vaccination at 14 dpv (Fig. 4A). Serum and colostrum were collected from sows to detect anti-PEDV antibody levels. Immunized sows showed no adverse reactions after vaccination. PEDV-S-conjugated IgG and IgA antibody titers in serum (Fig. 4B and D) and colostrum (Fig. 4C and E) were significantly elevated after immunization. IgG levels of anti-PEDV E, M, and N proteins in sow serum and colostrum (Fig. 4F and G) were also induced by VLP mRNA immunization. The neutralizing antibody titers against the different subtype PEDV strains of CH-HK-2021 (G2c subtype), AH2012/12 (G2b subtype), JS2008 (G1 subtype), and JS-NJ2023 (S-INDEL subtype) reached titers of 1:2^6^–2^8^ in serum (Fig. 4H), which exhibited a relatively good broad-spectrum neutralizing effect.

### Passive protection against PEDV in suckling piglets

**Fig 5:**
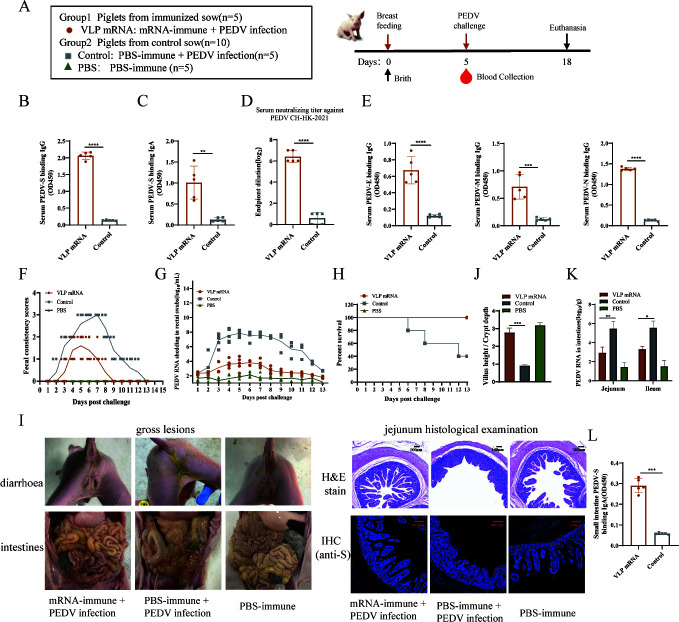
Passive protection against PEDV in suckling piglets. (**A**) Passive immunity challenge protocol: piglets were challenged with PEDV CH-HK-2021 on day 5 of lactation. (**B–D**) Passive antibody titers in serum collected on day 5 post-farrowing (pre-challenge) were measured for PEDV S-specific IgG, IgA, and neutralizing antibodies. (**E**) PEDV E, M, and N-specific IgG in serum was quantified by ELISA. (**F**) Diarrhea was scored daily, ≥2 indicated diarrhea. (**G**) Viral shedding of daily rectal swabs with PEDV-N gene copies per mL. (**H**) Survival percents of the piglets in different groups. (**I**) Gross and histopathology of the intestine tissues; PEDV-N (red), DAPI (blue); bar = 100 µm. (**J**) The Villus height/Crypt depth ratios of the jejunums. (**K**) Viral loads of the jejunums (mean ± SD; *P < 0.05, **P < 0.01, ***P < 0.001, ****P < 0.0001). (**L**) IgA levels of the small intestine of piglets.

The ability of the VLP mRNA vaccine to confer passive immunoprotection to suckling piglets was next investigated. After 5 days of lactation, piglets from immunized and control sows were challenged with PEDV (Fig. 5A). Antibody levels in piglet sera were determined, PEDV-S-specific IgG and IgA antibodies were significantly induced (Fig. 5B and C), and neutralizing antibody titers against the CH-HK-2021 strain reached approximately 1:2^6^ (Fig. 5D), whereas no specific antibodies were detected in the sera of piglets born to control sows, suggesting that serum antibodies were transferred through colostrum. Besides, the antibodies against PEDV N, E, and M proteins in serum of VLP mRNA immunized piglets were also increased (Fig. 5E). After the challenge, piglets born to control sows developed diarrhea at 2 dpc, which progressed to severe watery diarrhea and lasted for up to 10 days. However, piglets born to immunized sows started to have diarrhea at 4 dpc and maintained only mild diarrhea for approximately 5 days (Fig. 5F). PEDV RNA levels in the feces of piglets born to immunized sows were significantly lower than those of control piglets (Fig. 5G). No piglets died in the immunized group after challenge, whereas one piglet died in the control group on each of days 6, 8, and 12 following challenges (Fig. 5H).

The gross and histological lesions in piglets born to immunized and control sows after disease onset were analyzed. As shown in Fig. 5I, no significant gross lesions were found in the intestinal tissues of piglets born to immunized sows, whereas the intestinal tissues of piglets born to the control sows showed thinning of the intestinal wall, pneumatosis, and hemorrhage. Pathological examination showed no obvious histopathological changes in the ileum of piglets born to immunized sows, whereas piglets born to control sows showed obvious histopathological changes in the ileum, including blunted and atrophied villi, vacuolization, and disappearance of intestinal crypts and villous structures. Immunofluorescence analysis revealed obvious PEDV aggregation in the atrophied ileal villi of control piglets. In contrast, only sparse PEDV-positive cells were observed in the ileum of piglets from immunized sows. Moreover, the jejunal villus height-to-crypt depth ratio was significantly higher in piglets born to immunized sows than in the control group (Fig. 5J). PEDV RNA loads in the jejunum and ileum were notably higher in control piglets compared with those from immunized sows (Fig. 5K). Additionally, intestinal IgA levels were determined, and the results demonstrated significantly elevated IgA concentrations in the intestinal tissues of piglets born to immunized sows (Fig. 5L). These results suggest that the PEDV VLP mRNA vaccine confers effective passive immunity against PEDV challenge in newborn piglets.

## DISCUSSION

Since 2010, large-scale outbreaks of variant PEDV have caused substantial economic losses to the pig industry. Therefore, the development of a protective vaccine against PEDV remains a top priority. Owing to their high efficacy, rapid development, low manufacturing cost, and favorable safety profile, mRNA vaccines are expected to become an alternative to traditional approaches (30). Moreover, by leveraging the intrinsic advantages of VLPs, a VLP-forming SARS-CoV-2 mRNA vaccine has been developed that elicits antibodies of greater potency and breadth in mice than an mRNA vaccine encoding the S protein alone (31). Inspired by this, we designed a PEDV VLP-forming mRNA vaccine that co-expresses the four structural proteins S, E, M, and N and demonstrated that it provides superior immune protection compared with an S-only mRNA or conventional inactivated vaccines. Moreover, recent epidemiological studies have shown that the G2c subtype has become the dominant clinically prevalent PEDV strain (32). In this study, the CH-HK-2021 strain was used as the reference for vaccine design, and phylogenetic analysis of the complete S gene also confirmed that the CH-HK-2021 isolate belongs to the PEDV G2c subtype (Fig. S1) and is a currently clinically prevalent strain. Therefore, designing vaccines based on this strain is more clinically targeted and has greater application value.

Based on the validation results at the HEK293T eukaryotic cell, the VLP mRNA system can be correctly expressed and successfully assembled into VLPs *in vitro*, which confirmed the multi-component expression and assembly capabilities of the VLP mRNA. Therefore, for clinical application, experimental animals only require direct immunization via injection of the VLP mRNA vaccine. The processes of antigen expression and VLP assembly both occur *in vivo*, thereby inducing a more robust immune response in the host. This verifies the feasibility of the VLP mRNA vaccine and highlights its technical advantages in vaccine research, development, and application.

VLPs are particles self-assembled from viral structural proteins without any viral genome and are, therefore, non-infectious (33). Their morphology, size, and surface-antigen array closely mimic those of native virions, enabling them to simulate the infection process and elicit robust humoral and cellular immunity. Because the antigens are displayed in highly repetitive patterns (34), VLPs efficiently cross-link B-cell receptors, activate B cells, and induce strong antibody responses. They are also readily taken up by antigen-presenting cells such as dendritic cells and macrophages, which then process and present the antigens thereby through both MHC I and MHC II pathways to activate CD8^+^ and CD4^+^ T cells, generating a comprehensive immune response (35, 36). In this study, we found that the VLP mRNA vaccine elicited markedly higher levels of both humoral and cellular immunity, most likely attributable to the particulate structure of the VLPs and the inclusion of multiple viral structural proteins.

The PEDV E protein induces a pro-inflammatory immune response by activating endoplasmic reticulum stress and upregulating IL-8, thereby exerting an immunomodulatory role during viral pathogenesis (37, 38). The M protein plays a central role in regulating viral morphogenesis and assembly via multiple protein-protein interactions, including M-M, M-S, and M-N associations. Accumulating evidence has verified that the PEDV M protein can elicit the production of protective antibodies in pigs, while also being involved in antagonizing the cellular antiviral response orchestrated by type I and type III interferon signaling pathways (39–42). The N protein of PEDV is trafficked to the surface of infected cells via an exosome-dependent pathway. Although the resulting anti-N antibodies lack classical neutralizing activity, they can bind surface-exposed N protein and trigger antibody-dependent NK-cell-mediated cytotoxicity (ADCC), thereby eliminating infected cells through a non-neutralizing mechanism. Moreover, the N protein itself markedly enhances the secretion of IL-4 and IFN-γ, tilting the Th1/Th2 balance toward Th2 and promoting B-cell differentiation and antibody production. Collectively, the N protein not only “recruits” the ADCC arm of the antibody response but also amplifies humoral immunity through a cytokine network, highlighting its dual immunomodulatory role as an “antibody-response enhancer” (26, 43, 44). In contrast, the single S-subunit vaccine relies exclusively on the spike protein to elicit an immune response and lacks the innate immune-enhancing signals and multi-dimensional regulatory network provided by the E, M, and N proteins; consequently, it cannot effectively counteract viral interferon antagonism or establish potent and comprehensive immune activation. In this study, our VLP mRNA vaccine co-displays all four structural proteins—S, E, M, and N. Their concerted action markedly amplifies pattern-recognition receptor signaling, accelerates germinal center reactions, and preserves a high density of neutralizing epitopes, thereby eliciting neutralizing antibody and T-cell responses of greater titer, breadth, and durability, far surpassing the immunogenicity of S-only subunit vaccines. Actually, the cytokine analysis showed that all three immunized groups (VLP mRNA, S mRNA, and inactivated vaccine) induced IFN-γ production, whereas the VLP mRNA vaccine group also secreted high levels of IL-4. As IL-4 can inhibit Th1 cell differentiation and subsequent IFN-γ production (45), this may account for the lack of significant differences in IFN-γ levels among the three immunized groups. Meanwhile, the VLP mRNA vaccine simultaneously induced high levels of IFN-γ and IL-4, demonstrating a balanced ability to trigger Th1/Th2 immune responses and, thus, enabling more comprehensive and systemic activation of host immunity.

In the mouse immunization model, mRNA vaccines elicited high levels of IgG antibodies after prime immunization alone, with a significantly superior effect compared with the inactivated vaccine; this finding is consistent with previous studies (23). The underlying mechanism may involve the *in situ* translation of mRNA, which extensively activates innate immunity and accelerates adaptive immune responses (46–48). Such rapidly induced protective immunity is critical for defending against highly pathogenic viruses such as PEDV. In contrast, mucosal antibody titers were significantly increased only after booster immunization, suggesting that the establishment of mucosal immunity requires antigen transport to gut-associated lymphoid tissues (GALT) and completion of class-switch recombination, tissue-specific migration, and terminal differentiation of IgA-committed B cells (49, 50). These results also indicate that mucosa-targeting strategies should be incorporated into future vaccine design.

Passive immunity is a key strategy for protecting neonatal piglets against PEDV infection, and maternal antibodies can be transferred to piglets via colostrum and milk, thereby providing immediate protection during their most vulnerable period (16, 51). In this study, we observed that IgA levels in the serum and colostrum of pregnant sows were significantly elevated following booster immunization with the VLP mRNA vaccine. This result is consistent with our previous findings, which demonstrated that the mRNA vaccines induce stronger cellular immune responses and promote a significant increase in IgA levels than the conventional subunit vaccines and inactivated vaccines (16, 25). In addition, the unique virus-like structural properties of VLPs may further promote the activation of mucosal immune responses. It is speculated that the combined effect of the above two factors is an important reason for the high levels of IgA induced by the VLP mRNA vaccine, which also lays a foundation for piglets to obtain effective passive immune protection from sows. Furthermore, neutralization test results showed that sow serum could simultaneously neutralize different PEDV subtype strains, exhibiting the cross-protective potential of the vaccine. Such results indicate the broad-spectrum advantage of the PEDV G2c subtype strain as a vaccine candidate.

In this study, we designed and prepared a VLP mRNA vaccine capable of simultaneously expressing all four structural proteins of PEDV. We systematically evaluated its immunogenicity and protective efficacy against PEDV infection in piglets. The findings demonstrated that the mRNA vaccine encoding VLPs induced robust PEDV-specific humoral and cellular immunity in sows, offering effective protection for neonatal piglets through maternal antibody transfer. The mRNA platform offers four key advantages: high safety, rapid development, strong immunogenicity, and durable immune responses. VLPs, characterized by their native virion-like structure, highly repetitive epitopes display, and genome-free nature, exhibit excellent safety and immunogenicity. By integrating these two technologies through mRNA-encoded VLP, both neutralizing antibody levels and cellular immune responses can be further enhanced, thereby providing a practical and feasible technical pathway for the development of novel PEDV vaccines.

## Data Availability

All data have been included in the article and supplemental material. All reported data and any additional information required to reanalyze the data are available from the lead contact upon reasonable request.

## SUPPLEMENTAL MATERIAL

Fig. S1 jvi.02060-25-s0001.docx Phylogenetic tree of PEDV S gene sequences.

ASM does not own the copyrights to Supplemental Material that may be linked to, or accessed through, an article. The authors have granted ASM a non-exclusive, world-wide license to publish the Supplemental Material files. Please contact the corresponding author directly for reuse.
