# Supplementary material for: Development of a vaccine based on mRNA assembly of PEDV virus-like particle
Source: J Virol. 2026 Apr 21;100(5):e02060-25. doi: 10.1128/jvi.02060-25 (PMC13185548; doi:10.1128/jvi.02060-25)

**Fig. S1 Phylogenetic analysis of full-length S gene sequences from 51 PEDV strains**. Phylogenetic tree based on 47 S gene sequences of PEDV strains, including 4 isolates used in this study and other representative sequences retrieved from GenBank. The tree was constructed using MEGA 11 with 1000 bootstrap replicates. Isolates of G1a, G1b, S-INDEL, G2b, G2a and G2c subtypes are labeled in gray, yellow, purple, pink, blue and green, respectively.


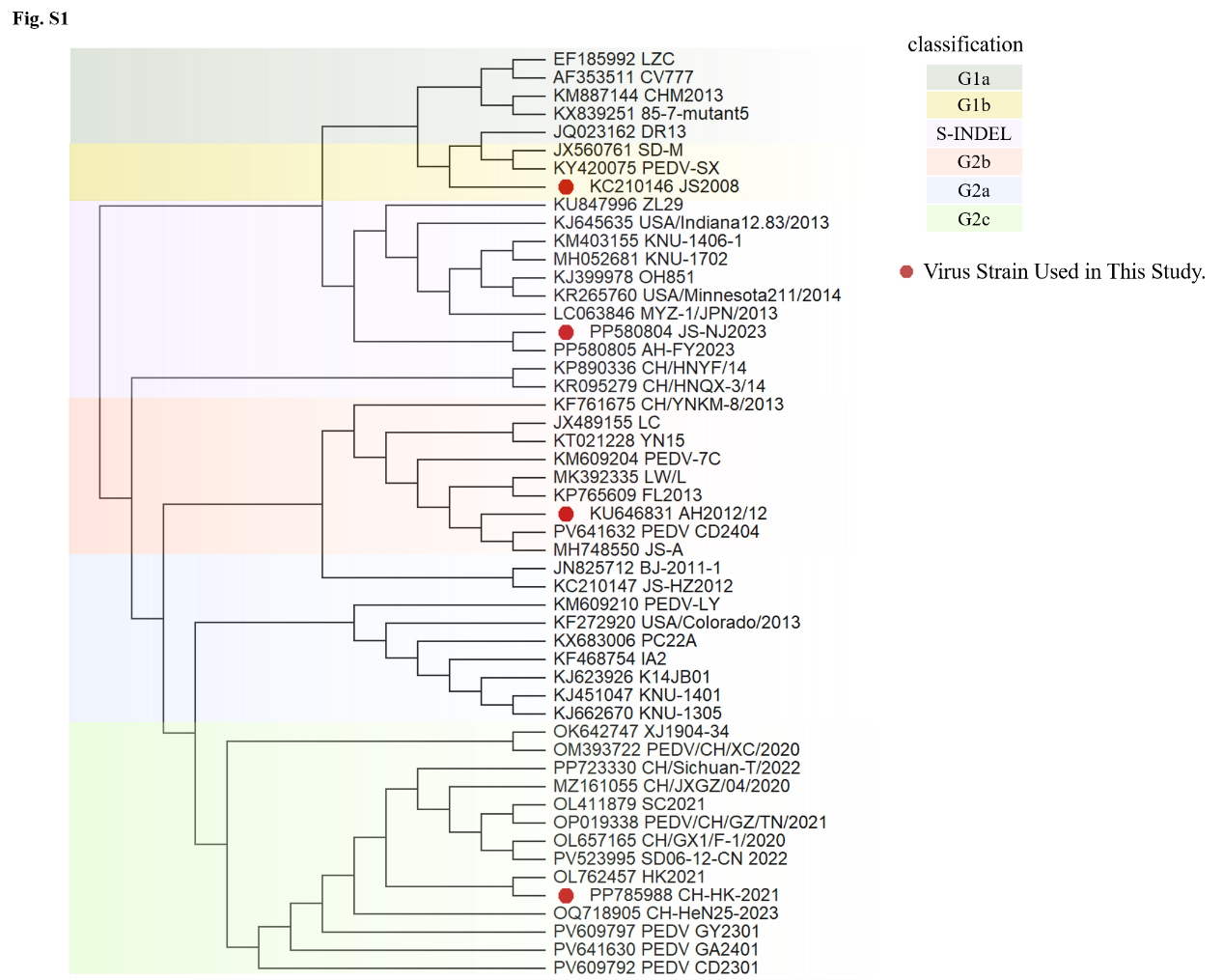

Supplement: Fig. S1 — Phylogenetic tree of PEDV S gene sequences. [file jvi.02060-25-s0001.docx]
